# Visible and subvisible particles in the BCG immunotherapeutic product ImmuCyst^®^

**DOI:** 10.1016/j.csbj.2016.03.001

**Published:** 2016-04-06

**Authors:** Marina Kirkitadze, Elena Remi, Kamaljit Bhandal, Bruce Carpick

**Affiliations:** Analytical Research & Development, Sanofi Pasteur, 1755 Steeles Avenue West, Toronto, Ontario, Canada

**Keywords:** Bacillus Calmette–Guerin (BCG), BCG immunotherapeutic product, ImmuCyst®, Electrical Sensing Zone method (ESZ), Characterization, Particle sizing

## Abstract

Bacille Calmette–Guerin, BCG, is a live attenuated bovine tubercle bacillus used for the treatment of non-muscle invasive bladder cancer. In this study, an Electrical Sensing Zone (ESZ) method was developed to measure the particle count and the size of BCG immunotherapeutic (BCG IT), or ImmuCyst® product using a Coulter Counter Multisizer 4® instrument. The focus of this study was to establish a baseline for reconstituted lyophilized BCG IT product using visible and sub-visible particle concentration and size distribution as reportable values. ESZ method was used to assess manufacturing process consistency using 20 production scale lots of BCG IT product. The results demonstrated that ESZ can be used to accumulate product and process knowledge of BCG IT.

## 1 Introduction

### 1.1 Bacillus of Calmette and Guerin (BCG)

*Mycobacterium bovis* (*M. bovis*) is a bacterium that causes tuberculosis in cattle and may also infect and cause illness among other animals, including humans. In humans, *M. bovis* affects lungs, lymph nodes, and other parts of the body. The original *M. bovis* Bacillus Calmette Guérin vaccine strain has developed into several different sub-strains which have been used for the production of BCG vaccines since 1921, and is used to immunize against tuberculosis since 1948 [1], [2], [3]. Beginning in the 1950s, cancer immunologist Lloyd J. Old and other researchers at Sloan Kettering began investigating BCG as a treatment for cancer [4], and subsequent clinical studies demonstrated the effectiveness of this therapy for early-stage bladder cancer [5], [6]. BCG has been used for treatment of non-muscle invasive bladder cancer since the early 1990's. The product referred as BCG Immunotherapeutic (BCG IT), or ImmuCyst® is a live attenuated bacterium; thus particles are inherent part of this product and may be important for immunogenicity. It is therefore informative to assess particle size as a potential quality attribute. In this work for the first time we present characterization of visible and subvisible particles in the BCG IT product, ImmuCyst®.

### 1.2 Purpose and scope of the study

Reconstituted lyophilized BCG IT product represents a heterogeneous suspension of particles with sizes in the visible and the sub-visible ranges. A suitable method was required for particle size and distribution of reconstituted lyophilized BCG IT suspension, in order to gain knowledge about (a) the product and (b) the performance of the manufacturing process.

Previously, a Laser Diffraction method was used to characterize size distribution of BCG IT products, while an Electrical Sensing Zone (ESZ) method was used for particle count, i.e. number of particles per mL. The design of the Coulter Counter Multisizer 4® instrument allows sample measurement in a confined chamber that prevents potential spread of aerosols in the laboratory. Since BCG IT is a live attenuated bacterium, this design was preferred over that of the Mastersizer 3000 instrument, in which the wet dispenser is not able to prevent aerosol release during the sample introduction step. Accordingly, an ESZ method was developed to measure both the particle count and the size distribution of BCG IT product, using a Multisizer 4 instrument.

Three production scale BCG IT lots were used for ESZ method development and optimization experiments, including a study to assess method precision. In order to support future process consistency and comparability studies, 20 additional production scale lots representative of the manufacturing process were used to establish a baseline set of ESZ data. Such data sets can provide information about the performance of the manufacturing process, as well as support future lot comparability studies [7]. In addition, it is useful to assess particle size and distribution as a potential product quality attribute. Unlike most small molecule and biotherapeutic products, many vaccines contain visible and sub-visible particles as endogenous components. Furthermore, the particulate nature of vaccines and other immunogenic formulations is well known to impact the host immune response. Thus, particle size and distribution is an interesting parameter to investigate from the perspective of lot consistency and product knowledge. In this study, the particle size and concentration measured by ESZ method for the lots of BCG IT product ImmuCyst® is described for the first time.

The scope of this study was limited to the Connaught strain only, as it is used to manufacture BCG IT product, ImmuCyst®. Other strains may produce particles of different size compared to Connaught. While it would be interesting to look at the particle size distribution of other BCG strains, such studies would require equivalent processing of the strain as per our current manufacturing process.

### 1.3 Analysis of particle dispersion using ESZ

ESZ is a method to determine the number and size of particles suspended in an electrolyte by causing them to pass through a small orifice in an electric field. Particles suspended in a weak electrolyte solution are drawn to a small aperture separating two electrodes through which an electric current flows. The voltage applied across the aperture creates a “sensing zone”. As each particle passes through the aperture it displaces is own volume of conducting liquid, momentarily increasing the impedance of the aperture. The changes in electrical impedance as particles pass through the orifice generate voltage pulses whose amplitudes are proportional to the volumes of the particles. The pulses are amplified, sized and counted and from the derived data the size distribution and particle concentration of the suspended phase can be determined. The method allows to determine size and concentration for particles from submicron to visible size (~ 400 nm to 1600 μm) depending on the system, and multiple apertures are required to cover this size range.

The method was originally applied to blood cell counting [8], [9], [10]. Kubitschek [11], [12], [13] introduced modifications which permitted counting of bacterial cells, and pointed out that this principle could be applied to the measurement of cell-volume distributions as well as number counting. Modified instruments were soon developed with which particles could be sized as well as counted, e.g. Multisizer 4 (Beckman Coulter).

An advantage of ESZ is that it does not depend on optical properties of particles and formulations. As such, it is a useful complementary technique to light based particle sizing techniques (e.g. laser diffraction, light obscuration, and flow imaging microscopy).

## 2 Materials and methods

### 2.1 BCG IT sample preparation

The BCG IT product is based on the Connaught sub-strain, which originated from the BCG parent strain of the Pasteur Institute in the late 1920s [3]. All three BCG IT lots were manufactured by Sanofi Pasteur, Toronto, Ontario, Canada and were lyophilized and sealed in glass vials. Typically, saline is used as diluent for BCG IT prior to patient administration. In this study, ISOTON® II electrolyte solution (Beckman Coulter) which contains 0.9 M NaCl in water was used as a diluent. Each BCG IT lot was dissolved in 3 mL of ISOTON® II and stirred continuously using a small magnet placed inside each vial on a stirring plate.

Particle counts and size distributions were measured by ESZ using a Coulter Counter Multisizer 4 instrument (Beckman Coulter, Brea, CA, USA). The particle size distribution of the reconstituted lyophilized BCG IT product was reported using derived diameters: d10, d50, and d90, whereas the concentration of the sub-visible particles was expressed as number of particles per mL. The dispersion of BCG IT suspension for 20 representative lots was expressed by the span, which is defined as (d90–d10)/d50, and is indicative of the polydispersity of the sample [14].

### 2.2 ESZ procedure

ESZ was performed using Beckman Coulter Multisizer 4® instrument (Beckman Coulter, Brea, CA, USA) equipped with the Multisizer4 software. A 280 μm aperture and a 1000 μm aperture were calibrated using 90 μm standard latex beads (Beckman Coulter). Each aperture is calibrated during vendor installation. The calibration records are retained by the software and can be retrieved for subsequent uses. For the particle count and size distribution measurements, a clean ST Beaker® (Beckman Coulter) was filled with 400 mL ISOTON® II electrolyte solution. Sample preparation and handling was performed aseptically in a laminar flow biocontainment cabinet. A 1.0 mL aliquot of stirred BCG IT sample was added to the solution in the 400 mL ST Beaker® and stirred in the Multisizer 4® during the entire experiment. For the size distribution measurement only to detect larger particles with the 1000 μm aperture, a clean ST Beaker® was filled with 400 mL of ISOTON® II electrolyte solution and glycerol 6:4 mixture. A 1 mL aliquot of stirred BCG IT sample was added to the 400 mL ST Beaker® and stirred in the Multisizer 4® during the entire experiment.

As discussed above, the analysis was done using two apertures, 280 μm and 1000 μm to allow full coverage of the particle size distribution. In general, the events of aperture blockage, low current, air in the aperture, the analyst is alerted, and the experiment is discontinued. In addition, in the event of blockage the particle concentration detected would be much lower than reported. In case of the 280 μm and 1000 μm apertures, no blockage was observed during the run. However, in case of 1000 μm aperture that consumes most of the sample during the run, a 90 s acquisition time was set to avoid air getting into the system.

### 2.3 Reportable values

The particle size distribution of the reconstituted lyophilized BCG IT product was reported using derived diameters: d10, d50, and d90, whereas the concentration of the sub-visible particles was expressed as number of particles per mL. The dispersion of BCG IT suspension for 20 representative lots was expressed by the span, which is defined as (d90–d10)/d50, and is indicative of the polydispersity of the sample [14].

## 3 Results and discussion

### 3.1 Development and optimization of the ESZ method

The purpose of ESZ method development and optimization was to demonstrate that this method is appropriate to measure the size distribution of visible and sub-visible particles, and count of sub-visible particles in BCG IT samples. The scope of ESZ method development and optimization included the choice of sample lots, sample preparation, instrument parameters, and an assessment of precision. The precision of the ESZ method was evaluated through a lot-to-lot and analyst-to-analyst study, using the same instrument parameters, sample preparation, data collection and analysis procedures. The derived diameters, d10, d50, and d90, and particle concentrations from the different runs were collected and analyzed statistically to assess precision.

Normally one representative product lot is sufficient for a method development study. However, in this case three in-house non-commercial lots representative of BCG IT lyophilized filled product were used to develop the sample preparation procedures and Multisizer 4 instrumental parameters suitable to measure particle concentration and size distribution.

Under the assumption that a sub-population of BCG IT particles fell within the visible range, an aperture of 1000 μm was initially selected to detect the larger particles. An aperture of 280 μm was used to measure particle concentration and size distribution in the range 5–168 μm. The measurements of particle concentration and size distribution were performed by two analysts for the three reconstituted BCG IT lots as described above under the heading “ESZ Procedure”. All BCG IT samples were reconstituted and stirred directly in the sample vial to ensure a uniform suspension of the particles. An aliquot of a continuously stirred BCG IT sample was then taken and diluted 1:400 in the electrolyte solution for the ESZ analysis. The length of experiment, 90 s, was determined as the time at which the level of the analyte is just above the orifice of the aperture; this corresponded to approximately 300 mL of analyte solution aspirated by either the 280 μm or 1000 μm aperture. The precision for measuring particle count and size distribution of BCG IT vaccine was assessed by evaluating lot-to-lot and analyst-to-analyst variability.

**Fig 1:**
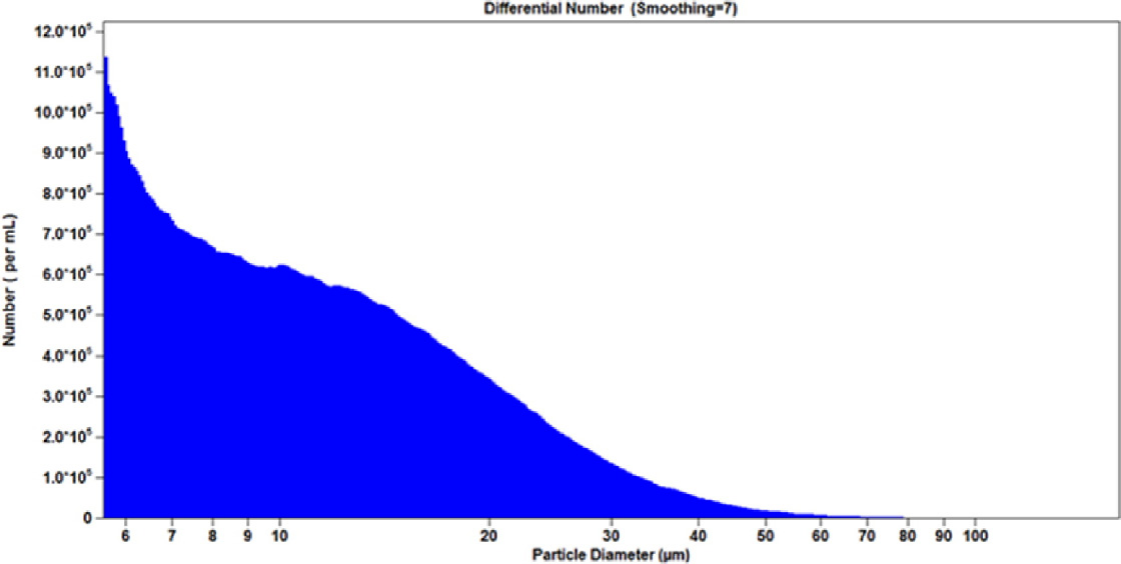
Concentration of sub-visible particles according to their size in BCG—IT sample. Sub-visible particle concentrations, reported as number per mL, according to their size, in μm, were measured by the 280 μm aperture. As the graph illustrates, smaller particles are more prominent in reconstituted BCG IT samples.

**Fig 2:**
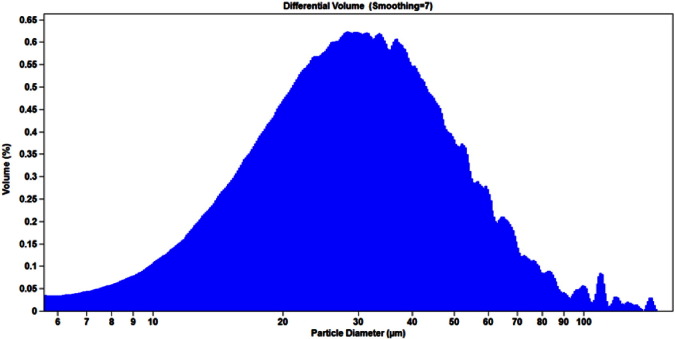
Size distribution of particles in BCG IT sample measured by the 280 μm aperture. Visible and sub-visible particle size distribution in reconstituted BCG IT product was measured by the 280 μm aperture and reported as percentage of volume analyzed through the aperture. By % volume, the size distribution has a bell-curve distribution that peaks around 26 μm.

**Fig. 3:**
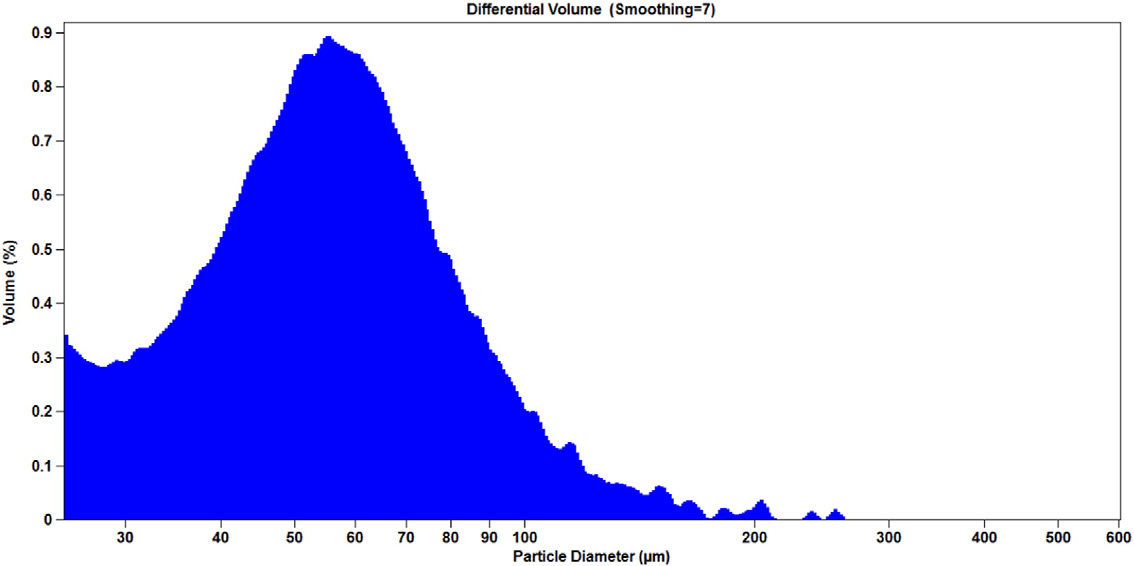
Size distribution of particles in BCG IT sample measured with the 1000 μm aperture. Visible and sub-visible particle size distribution in reconstituted BCG IT product measured by the 1000 μm aperture and reported as percentage of volume analyzed through the aperture. By % volume, the size distribution is a left-skewed bell-curve that peaks around 56 μm.

Examples of particle concentration and size distribution profiles are shown in Fig 1, Fig 2, Fig. 3. The results obtained for three representative lots of BCG IT are summarized in Table 1, Table 2.

The observed particle count and size distribution profiles were consistent for the three lots of BCG IT product (Table 1, Table 2). The %CV value obtained for the particle concentration expressed as number per mL was 2.5%. The values of calculated %CV for precision of derived diameter measurements using the aperture of size 280 μm were 6.2%, 8.7%, and 9.4% for d10, d50, and d90 respectively. The %CV values for derived diameters measured by the aperture of size 1000 μm were higher, 7.9%, 11.7%, and 21.8% for d10, d50, and d90 respectively.

Accordingly, the ESZ method was judged to be appropriate for measuring particle count and concentration of BCG IT using the 280 μm aperture, based on the overall lower %CV values obtained with two runs over the three lots. In addition, as shown in Fig. 3, a population of the particles below 25 μm in diameter was not detected using the aperture of size 1000 μm. In contrast, the aperture of size 280 μm detects the entire population of the BCG IT particles in the range of 5–168 μm (Fig. 2).

Independent experiment (results not shown) using Mastersizer 2000 demonstrated that three lots BCG IT lots showed the Dv50 in the range of 19.9–45.7 μm. The variability was greater from run to run for the same lot. The samples were reconstituted in 0.9% saline, and dispensed in Milli Q water, and measured at the mixing speed of 2205 rpm.

Moreover, use of the 280 μm aperture for both the particle count and size distribution measurements reduces both the overall experimental time, and the amount of BCG IT sample required for the measurement. Based on the results of this study, it was thus decided to use the 280 μm aperture for the baseline assessments of the multiple production scale BCG IT lots.

### 3.2 Qualification of ESZ method

Assay qualification was performed to demonstrate that the method is scientifically sound and suitable for its intended use. The qualification study consisted of a method precision assessment. The precision for measuring particle concentration and size distribution of BCG IT vaccine was assessed by evaluating day-to-day and analyst-to-analyst variability. The qualification study was performed using one lot, and included three repeats of particle concentration (i.e. number per mL) determination, and size distribution parameters d0, d50, d90 collected during by two analysts over three days. The span was used to describe distribution width. In total, there were nine measurements in total for both particle concentration and size distribution (Table 3).

Repeatability was assessed using three reportable values generated by the same analyst within the same day. The Coefficient of Variation (CV) in % was calculated for the three reportable values as per Eq. (1): %CV = (standard deviation ÷ mean) × 100%. Repeatability was assessed for each analyst-day combination; therefore, there are three %CV values reported per reportable value. Intermediate precision was assessed by having two analyst performing a total of 9 independent measurements for 3 days (9 reportable values) using the samples of the same lot of BCG IT. The %CV was calculated based on the 9 measurements (Table 4).

The intermediate precision and repeatability %CV were below 3% for all reportable values (Table 4). There is no pre-defined desirable method performance for this qualification study, the low %CV values obtained were judged to be acceptable. Qualification of the ESZ procedure for BCG IT demonstrated that the method is suitable for its intended purpose, and will be used to support comparability studies of the BCG IT product [15], [16].

### 3.3 Creating a baseline for the manufacturing process

Twenty lots of BCG IT were manufactured in order to confirm that the process at full scale can be run according to the established product specifications, up to the lyophilized product stage. These lots were performed at full scale in the existing manufacturing facility.

**Fig. 4:**
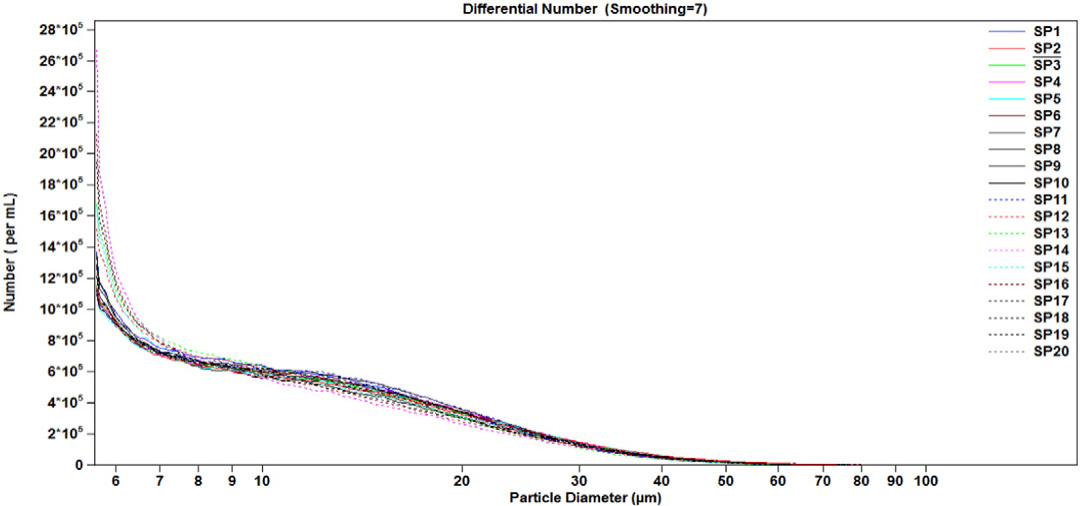
Sub-visible particles concentration in the 20 lots of BCG IT Concentration of sub-visible particles was measured on 20 production-scale lots of BCG IT to build an empirical baseline for the manufacturing process.

**Fig. 5:**
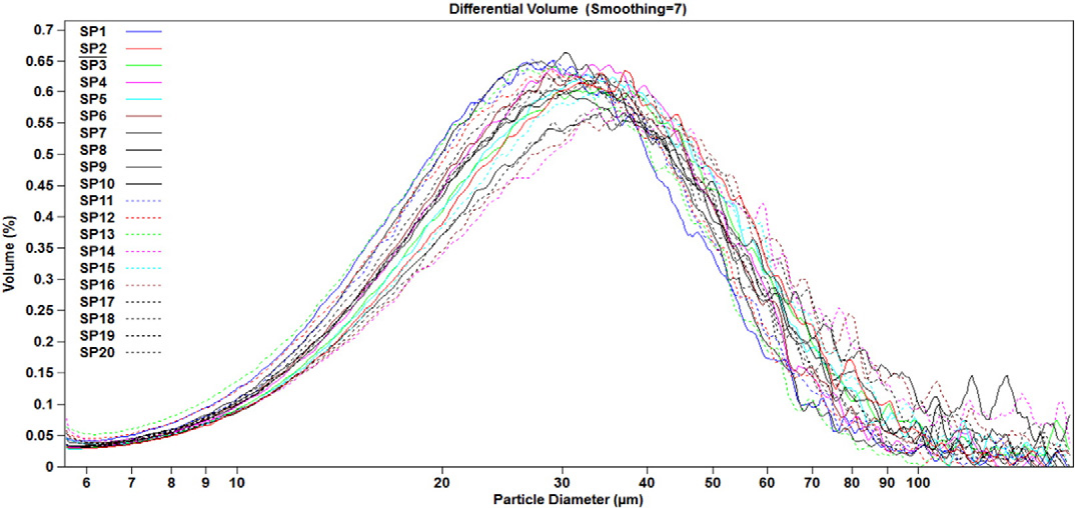
Particle size distribution in the 20 lots of BCG IT Particle size distribution profiles were collected for 20 production-scale lots of BCG IT to build an empirical baseline for consistency of the manufacturing process.

Process consistency for these lots was assessed from the standpoint of particle concentration and size distribution in BCG IT reconstituted lyophilized suspension. To address these parameters, ESZ reportable values were collected using the 280 μm aperture for the 20 BCG IT lots (Fig. 4, Fig. 5, Table 5).

The derived diameters and particle concentrations for the 20 lots of BCG IT are summarized in Table 5, and were consistent across the 20 lots analyzed. For particle concentration, derived diameters d10 and d50, the coefficients of variation for all measurements were below 10%, whereas for d90, %CV was slightly higher, at 13.8%. While the %CV from these 20 BCG IT lots are slightly higher for d90 and particle concentration compared to the ones collected from the three non-commercial BCG IT lots, the results nevertheless demonstrate lot-to-lot consistency between representative manufactured lots.

The dispersion of BCG IT suspension, as expressed by the span (Table 5), was 1.54 on average, with %CV of 8.8. This shows that the overall dispersion for BCG IT suspension was consistent for the 20 representative lots.

Overall, these results confirm that ESZ is an appropriate method for measuring particle concentration and size for BCG IT vaccine.

## 4 Conclusions

The analysis of the particle count and size distribution results for BCG IT during method development, optimization, and qualification showed that the ESZ method was appropriate for measuring particle concentration and size distribution. Therefore, this method was selected to assess the consistency of the manufacturing process over 20 production scale lots of BCG IT.

ESZ can be used to measure both particle count and size distribution of BCG IT using the aperture of size 280 μm in the same instrument, reducing overall experimental time and sample volume requirements. The use of a single method (ESZ) for measurement of both particle count and size distribution simplifies the characterization package as this was previously done using two methods, Laser Diffraction for size distribution and ESZ for particle count.

In addition, the design of Coulter Counter Multisizer 4® requires a lower amount of the diluent (electrolyte) compared to a typical Laser Diffraction instrument (e.g. Mastersizer series 2000 and 3000), and fewer cleaning steps required due to the use of electrolyte solution (ISOTON II®) that prevents adsorption of samples to the aperture and other parts of the instrument.

Qualification of the ESZ procedure for BCG IT demonstrated that the method is suitable for its intended purpose, and will be used to support comparability studies of the BCG IT product [15], [16].

For any biological product, a characterization package consisting of relevant analytical methods will expedite product development through gaining of product knowledge, assessing lot-to-lot consistency and the impact of process change and identifying potential product quality attributes [17]. ESZ is an instrument-based method that does not need any alterations to the sample, which is especially important at the final product stage of manufacturing. In addition, ESZ can be performed on complex formulations, such as adjuvanted vaccines as well as products with visible and/or sub-visible particles as endogenous components. As a part of a test package for investigational products, ESZ offers considerable advantages as a method for particle count and size distribution assessment.

Considering the importance of BCG IT for clinical use such as intravesical immunotherapy in superficial bladder cancer patients and the fact that its production was developed approximately 20 years ago, development of new techniques that could support potential modernization of the manufacturing process of BCG IT would have great clinical importance.

## Executive summary

*Background*

• An electrical sensing zone (ESZ) method development, optimization, and qualification are described, with specific focus on Immucyst®, a BCG IT product characterization.

*Method development study design*

• Aspects to consider in development study: purpose and scope of the analytical procedure, product type, experimental design, and data analysis including the use of statistical methods.
• Aspects of ESZ method development and optimization are discussed, including rationale, reporting values, desirable performance, and characteristics (in this case, precision and lot-to-lot consistency).

*Results and discussion*

• ESZ method development is presented to characterize visible and sub-visible population of particulates present in BCG IT lyophilized reconstituted product.

*Conclusions & recommendations*

• Successful completion of the development study provides scientific evidence that the method is suitable for characterization of BCG IT reconstituted lyophilized product. Further qualification can also provide guidance and useful information for the eventual method validation, where required.

## Conflict of interest

The authors are employees of Sanofi Pasteur.

The authors have no relevant affiliations or financial involvement with any organization or entity with a financial interest in or financial conflict with the subject matter or materials discussed in the manuscript. Thus includes employment, consultancies, stock ownership or options, or royalties.

No writing assistance was utilized in the production of this manuscript.

## Figures and Tables

**Table 1:** Particle concentration and derived diameters for three BCG IT lots using the 280 μm aperture.

Trial lot (T)	Mean, μm	Concentration, number per mL	d10, μm	d50, μm	d90, μm
T1 1st run	11.5	10.7 × 10^**7**^	11.4	23.0	42.9
T1 2nd run	11.5	11.5 × 10^**7**^	11.3	22.9	42.8
T2 2 1st run	11.9	11.0 × 10^**7**^	12.5	26.8	50.4
T2 2 2nd run	11.9	11.0 × 10^**7**^	12.5	26.6	49.8
T3 3 1st run	12.2	10.9 × 10^**7**^	13.0	27.7	52.5
T3 3 2nd run	12.2	10.8 × 10^**7**^	13.0	27.8	53.0
Average	**11.87**	**11.0** × **10**^**7**^	**12.28**	**25.80**	**48.57**
Std. Dev.	**0.31**	**0.28** × **10**^**7**^	**0.76**	**2.26**	**4.59**
%CV	**2.6**	**2.5**	**6.2**	**8.7**	**9.4**

The average, standard deviation, coefficient of variation were shown in bold.

**Table 2:** Derived diameters of three lots BCG lots using the 1000 μm aperture.

Lot	d10, μm	d50, μm	d90, μm
T1 1st run	29.7	50.5	114.0
T1 2nd run	29.6	48.9	81.6
T2 2 1st run	35.3	64.6	136.4
T2 2 2nd run	35.4	62.7	119.6
T3 3 1st run	32.2	52.2	82.2
T3 3 2nd run	32.6	55.1	89.8
Average	**32.5**	**55.7**	**103.9**
Std. Dev.	**2.55**	**6.54**	**22.68**
%CV	**7.9**	**11.7**	**21.8**

The average of derived diameters were shown in bold.

**Table 3:** ESZ qualification study — summary of results for one lot of BCG IT.

		d10 (μm)	d50 (μm)	d90 (μm)	Span	Concentration (# per mL)
Day 1, analyst 1	Run 1	13.33	27.68	53.02	1.434	1.087 × 10^8^
	Run 2	13.14	26.92	50.73	1.396	1.095 × 10^8^
	Run 3	12.66	26.52	51.16	1.452	1.096 × 10^8^
Day 2, analyst 2	Run 1	12.99	27.02	51.10	1.410	1.090 × 10^8^
	Run 2	12.94	26.96	51.61	1.434	1.090 × 10^8^
	Run 3	13.00	26.94	51.73	1.438	1.089 × 10^8^
Day 3, analyst 1	Run 1	13.43	27.86	53.37	1.434	1.088 × 10^8^
	Run 2	13.15	27.49	52.11	1.417	1.087 × 10^8^
	Run 3	13.16	27.70	53.70	1.464	1.087 × 10^8^
Average		**13.09**	**27.23**	**52.06**	**1.431**	**1.090** **×** **10**^**8**^

The average of derived diameters, span, and concentration were shown in bold.

**Table 4:** ESZ qualification study — statistical analysis of results for one lot of BCG IT.

Variable	Repeatability (%CV)				Intermediate precision (%CV)
	Run 1	Run 2	Run 3	Overall	
d10	2.65%	0.25%	1.20%	1.36%	1.73%
d50	2.18%	0.15%	0.67%	1.00%	1.69%
d90	2.36%	0.65%	1.58%	1.53%	2.05%
Span	1.98%	1.04%	1.63%	1.55%	1.43%
Concentration	0.45%	0.05%	0.05%	0.19%	0.31%

**Table 5:** Particle concentration and derived diameters for 20 BCG lots using the 280 μm aperture.

Lot	Concentration, number per mL	d10, μm	d50, μm	d90, μm	Span (d90-d10)/d50
1SP	10.7 × 10^7^	14.5	34.6	76.2	1.78
2SP	10.7 × 10^7^	14.6	34.4	72.7	1.69
3SP	12.7 × 10^7^	14.0	32.3	64.4	1.56
4SP	11.2 × 10^7^	13.1	28.4	54.6	1.46
5SP	11.2 × 10^7^	12.8	27.0	52.2	1.46
6SP	10.8 × 10^7^	14.5	32.4	64.4	1.54
7SP	12.4 × 10^7^	14.6	33.5	70.1	1.66
8SP	10.4 × 10^7^	13.9	29.9	56.8	1.43
9SP	10.6 × 10^7^	14.1	30.0	59.5	1.51
10SP	10.7 × 10^7^	13.8	29.0	56.7	1.48
11SP	10.9 × 10^7^	13.6	28.1	53.5	1.42
12SP	10.6 × 10^7^	14.1	30.4	63.1	1.61
13SP	10.1 × 10^7^	13.8	29.9	59.8	1.54
14SP	10.9 × 10^7^	13.5	27.8	51.5	1.37
15SP	10.4 × 10^7^	14.7	33.4	78.5	1.91
16SP	10.6 × 10^7^	14.0	29.6	56.7	1.44
17SP	10.5 × 10^7^	14.6	31.4	60.9	1.47
18SP	10.6 × 10^7^	14.2	29.9	56.0	1.40
19SP	10.5 × 10^7^	14.5	31.4	62.6	1.53
20SP	10.4 × 10^7^	14.7	32.0	62.0	1.48
Average	**10.8** × **10**^**7**^	**14.0**	**30.6**	**61.9**	**1.54**
Std. Dev.	**0.66** × **10**^**7**^	**0.55**	**2.49**	**8.55**	**0.14**
%CV	**6.14**	**3.91**	**8.16**	**13.81**	**8.81**

The average of concentration, derived diameters, and span were shown in bold.
